# Depressive Symptoms Are Not Associated with Leukocyte Telomere Length: Findings from the Nova Scotia Health Survey (NSHS95), a Population-Based Study

**DOI:** 10.1371/journal.pone.0048318

**Published:** 2012-10-25

**Authors:** Jonathan A. Shaffer, Elissa Epel, Min Suk Kang, Siqin Ye, Joseph E. Schwartz, Karina W. Davidson, Susan Kirkland, Lawrence S. Honig, Daichi Shimbo

**Affiliations:** 1 Center for Behavioral Cardiovascular Health, Columbia University, New York, New York, United States of America; 2 Department of Psychiatry, University of California San Francisco, San Francisco, California, United States of America; 3 Taub Institute for Research on Alzheimer's Disease and the Aging Brain, Columbia University, New York, New York, United States of America; 4 Department of Psychiatry and Behavioral Science, Stony Brook University, Stony Brook, New York, United States of America; 5 Department of Community Health and Epidemiology, Dalhousie University, Halifax, Nova Scotia, Canada; 6 Gertrude H. Sergievsky Center, Columbia University, New York, New York, United States of America; 7 Department of Neurology, Columbia University, New York, New York, United States of America; University of Medicine and Dentistry of New Jersey, United States of America

## Abstract

**Background:**

Premature shortening of leukocyte telomere length has been proposed as a novel mechanism by which depression may confer increased risk of adverse cardiovascular events. Prior studies demonstrating associations of depression and depressive symptoms with shorter leukocyte telomere length were small, included selected psychiatric outpatients, were based on convenience samples, and/or adjusted for a limited number of possible confounding factors.

**Methods and Findings:**

We examined the associations of depressive symptoms, probable depressive disorder, and specific depressive symptom clusters, as assessed by the Center for Epidemiological Studies—Depression (CES-D) scale, with leukocyte telomere length, measured by using a real-time PCR method, in 2,225 apparently healthy participants from the 1995 Nova Scotia Health Survey population-based study. The mean age was 48.2±18.9 years; 49.9% of participants were female; and the mean CES-D score was 7.4±7.9. The mean telomere length was 5,301±587 base pairs. In an unadjusted model, depressive symptoms were significantly associated with longer leukocyte telomere length (B = 27.6 base pairs per standard deviation increase in CES-D, 95% confidence interval [CI] = 3.1–52.1, *p* = 0.027). This association was no longer significant after adjustment for age and sex (B = 9.5, 95% CI = −14.6–33.6, *p* = 0.44) or after further adjustment for body mass index, Framingham risk score and previous history of ischemic heart disease (all *p*'s≥0.37). Neither probable depressive disorder nor specific depressive symptom clusters were independently associated with leukocyte telomere length.

**Conclusions:**

Concurrent depressive symptoms were not associated with leukocyte telomere length in a large, representative, population-based study.

## Introduction

Depressive symptoms and depressive disorders are important causes of disability worldwide. [1] Depression has also been found to predict incident and recurrent cardiovascular disease (CVD) events and mortality [2], [3]; however, the pathophysiology and mechanisms underlying the association between depression and CVD events remains poorly understood. Accelerated cellular aging has been proposed as a novel pathogenic process associated with depression [4] and a biological mechanism by which depressive symptoms may confer increased risk of adverse cardiovascular events due to the role of vascular cellular aging in atherosclerosis development [5]. Telomeres are repetitive, non-coding DNA sequences located at the end of chromosomes that promote genome stability by protecting against aberrant DNA repair mechanisms. Telomere length has been proposed as a biomarker for biological aging [6], and shorter leukocyte telomere length has been associated with a variety of age-related disorders, including atherosclerosis [7], cardiovascular diseases [8], [9], dementia [10], and cancer [11].

Perceived stress and chronicity of stress have both been linked to shorter leukocyte telomere length in premenopausal women [12]. Following an early study by Simon and colleagues that reported significantly shorter leukocyte telomere length in a clinical sample of 44 patients with chronic mood disorders compared to 44 age-matched controls without psychiatric disorders [4], some studies have found an association of depressive symptoms and major depressive disorder (MDD) with shorter leukocyte telomere lengths [13], [14], while others have not [15], [16]. The enrollment of selected participants such as psychiatric patients, small sample sizes, and adjustment for a limited number of possible confounding factors including cardiovascular risk factors may have contributed to these divergent results.

We examined the association between depression and leukocyte telomere length in 2,225 participants from the population-based 1995 Nova Scotia Health Survey study, and determined whether this association was independent of age, sex, body mass index and other cardiovascular risk factors. Given that depression is a complex phenotype [17] and that specific depressive symptom clusters may be differentially related to cardiovascular prognosis [18] and biological factors related to cardiovascular prognosis [19], [20], we secondarily investigated whether specific depressive symptom clusters are differentially associated with shorter leukocyte telomere length.

## Materials and Methods

### Study population

The 1995 Nova Scotia Health Survey (NSHS95) is a representative, population-based survey implemented by Heart Health Nova Scotia and the Nova Scotia Department of Health. Potential participants were randomly identified based on a probability sample by Statistics Canada, the national statistical agency and census bureau, and were selected to be representative of the Nova Scotian population by age, sex, and geographic location. Participants consisted of non-institutionalized, non-pregnant Nova Scotians, ages 18 years or older, and listed in the registry of Medical Services Insurance, the government-sponsored universal health insurance plan. Of 4,500 targeted participants, 3,227 (72%) provided informed consent and were enrolled. As previously reported [21], propensity score analyses revealed no meaningful response biases between those enrolling versus those not enrolling in the study. The current study and analyses were approved by the institutional review boards of Dalhousie University, Halifax, Nova Scotia, and Columbia University Medical Center, New York, NY, and conform to the ethical guidelines of the Declaration of Helsinki. Participants provided written informed consent to participate in this study, which allowed for linkage to their health care use data and for the storage and future use of blood assays.

For this analysis, we excluded 1,002 participants who had depressive symptom scores but did not have assessments of leukocyte telomere length (*n* = 938), had assessments of leukocyte telomere length but were missing assessments of depressive symptoms (*n* = 17), or had no assessment of either measure (*n* = 47). Therefore, a final cohort of 2,225 participants was available for the present analysis. Compared to the 1,002 participants who did not have assessments of leukocyte telomere length or depressive symptoms, the 2,225 participants in this analysis were significantly less likely to be active smokers but did not differ significantly in age, sex, body mass index (BMI), systolic and diastolic blood pressure, or Framingham risk score (as defined below). Compared to the 938 participants who had depressive symptom scores but did not have assessments of leukocyte telomere length, the 2,225 participants included in the present analyses had significantly but modestly lower depressive symptom scores (mean±SD, 7.4±7.9 versus 8.3±8.5, respectively, *p* = 0.004).

### Nova Scotia Health Survey procedures

From March through November 1995, a group of trained public health nurses contacted potentially eligible individuals and interviewed those who agreed to participate. Those who were interviewed also visited a health care clinic approximately one week afterwards. During the clinic visit, height and weight were measured, a comprehensive set of cardiovascular risk factors was assessed, and a full medical history was conducted. Blood was drawn at the clinic visit and processed for plasma and

buffy coat samples. Medication use was recorded at a home visit.

### Measurement of leukocyte telomere length

DNA was extracted from frozen buffy coat samples. Leukocyte telomere length measurements were performed on coded samples by laboratory personnel blinded to participant characteristics. Average leukocyte telomere length was determined using a polymerase chain reaction (PCR) method modified from that of Cawthon and colleagues [9] by Kang and Honig (manuscripts in preparation). Real-time PCR was performed using a CFX384 thermocycler (Biorad, Richmond, CA). The assay method was optimized for use of both telomere (T) and single copy gene (S) amplifications on the same 384-well plate, with reference standard DNA samples on each plate. Test DNA samples each underwent two triplicate PCR reactions, with use of “calibrator samples” for correction of inter-plate variability. Amplification primers for telomeres included T_for_: 5′- CGGTTTGTTTGGGTT TGGGTTTGGGT TTGGGTTTGGGTT-3′ and T_rev_: 5′-GGCTTGCCTTACCCTTACCCTTAC CCTTACCCTTAC CCT-3′, and for single copy gene (beta-globin) S_for_ 5′- GCTTCTGACACA ACTGTGTTCACTA GC-3′ and S_rev_ 5′- CACCAACTTCATCCAC GTTCACC-3′. Thermocycling parameters were 95°C×10 min activation, followed by 34 cycles of 95°C×15 sec, and 55°C×120 sec. Assay coefficient of variance was 5% to 8%. Given that the T/S ratio depends on the particular DNA standards used, T/S ratios were converted to telomere base pairs using a formula (base pairs = (1,585 * T/S ratio)+3,582) derived from co-analysis of selected DNA samples with both PCR and terminal restriction fragment methods (non-radioactive TeloTAGGG Telomere Length, Roche Diagnostics, Mannheim, Germany). To allow comparisons between our study and others, we present telomere length results in base pairs. Given that the telomere base pairs are derived via a linear transform of the T/S ratio, all statistical analyses yield the same results regardless of whether base pairs or T/S ratio is used.

### Measurement of depressive symptoms

Depressive symptoms were measured using the Center for Epidemiological Studies—Depression (CES-D) scale [22], a 20-item self-report instrument designed for use in epidemiologic studies as a measure of current depressive symptoms. Analyses were performed using the scale as a continuous measure (Cronbach's alpha = 0.88 in this sample) and as a dichotomous measure, with cutpoints of CES-D≥10 or CES-D≥16 as markers of “elevated depressive symptoms” and “probable depressive disorder,” respectively. The CES-D≥16 cutpoint has been shown to have acceptable specificity and sensitivity in relation to interview-based diagnoses of MDD [23], [24]. We have also previously shown that elevated depressive symptoms, defined as CES-D≥10, are associated with an increased risk of ischemic heart disease events [25].

### Baseline demographic and risk factors

Demographic, anthropometric, and other cardiovascular risk factors that have been linked to leukocyte telomere length—age [26], sex [10], [27], elevated blood pressure [28], diabetes [29], increased BMI [30], [31], smoking status [31], and prevalent ischemic heart disease [32]—were assessed at baseline. Participants' ages and sex were recorded from the provincial health insurance registry and verified by the interviewer. Weight and height were measured twice, averaged, and used to calculate BMI (as weight in kilograms divided by height in meters squared). Registered nurses used manual sphygmomanometers with appropriately sized blood pressure cuffs to measure systolic blood pressure (SBP) and diastolic blood pressure (DBP); two readings at home and two at the clinic visit were averaged to determine resting values of each. Total cholesterol, high-density lipoprotein (HDL) cholesterol, and triglyceride levels were assayed from plasma samples by the Lipid Research Laboratory, University of Toronto, Ontario [33]. Low-density lipoprotein (LDL) cholesterol was calculated using the Friedewald formula [34]. History of diabetes was ascertained by self-report. Those who reported smoking currently or in the past year were considered smokers; all others were considered nonsmokers. In order for an efficient use of CVD risk factors in adjusted models, Framingham risk score was calculated using age, sex, total and HDL cholesterol levels, blood pressure, and history of diabetes and cigarette smoking [35]. Lipid-lowering medication use was defined as the use of statins, fibrates, bile acid sequestrants, or nicotinic acid. Prior ischemic heart disease was defined from a search of hospitalization data five years prior to the baseline interview and based on International Classification of Disease (ICD-9) codes 410.x through 414.x. Additional details of study procedures have been published previously [36].

### Statistical analyses

We used SPSS version 18 (IBM, Armonk, NY, USA) to analyze the association of depressive symptoms and probable depressive disorder with leukocyte telomere length. To determine the unadjusted association between depressive symptoms and leukocyte telomere length, we conducted linear regression analyses with leukocyte telomere length as the dependent variable and depressive symptoms as the independent variable (first treated continuously and then as one of two dichotomous variables to indicate either elevated depressive symptoms (CES-D≥10) or probable depressive disorder (CES-D≥16). We then adjusted this model for potential clinical-demographic confounders of the putative telomere-depression association. Model 1 adjusted for age and sex; model 2 additionally adjusted for Framingham risk score; model 3 additionally adjusted for BMI; and model 4 additionally adjusted for previous ischemic heart disease. Effect size modification was assessed by checking for interactions between depressive symptoms and participant characteristics including age (≥75 years vs. <75 years), sex, BMI (<25 kg/m^2^, 25–29.9 kg/m^2^, ≥30 kg/m^2^), and Framingham risk score (>10% vs. ≤10%) using likelihood-ratio tests after interaction terms were added to the full model. Sensitivity analyses were conducted excluding those with prior ischemic heart disease.

Secondary analyses were conducted to test whether specific depressive symptom clusters were associated with leukocyte telomere length. To do so, we computed four subscales of the CES-D based upon previously reported factor analytic studies showing that this measure has a four-factor structure [37]. These four subscales were: depressed affect (items 3, 6, 9, 10, 14, 17, and 18), somatic concerns (items 1, 2, 5, 7, 11, 13, and 20), positive affect (items 4, 8, 12, and 16), and interpersonal problems (items 15 and 19). The set of five unadjusted and adjusted linear regression models discussed above were used to examine the association of leukocyte telomere length with each depressive symptom cluster, treated as a continuous measure. We additionally investigated whether high versus low scores on each subscale (defined as the fourth quartile versus the first quartile) was associated with leukocyte telomere length in a similar series of unadjusted and adjusted linear regression models.

### Power analysis

Simon and colleagues [4] reported that those with a mood disorder had significantly shorter leukocyte telomere lengths than a non-psychiatric comparison group, controlling for age and sex. From their regression analysis results, we calculated that the effect size for their observed difference in adjusted group means was *d* = −0.67, which corresponds to a partial point-biserial correlation of −0.33. The effect size *d* for the association of leukocyte telomere length and depression in other studies ranged from −0.83 to −0.59, corresponding to point-biserial correlations of −0.38 and −0.28, respectively. Although we hypothesize that the true correlation is negative, there is clearly substantial uncertainty as to the magnitude of the actual effect size. Nonetheless, with a sample of 2,225 participants, the present study had greater than 99% power to detect a partial correlation≤−0.10, using a two-tailed F-test of the corresponding regression coefficient for CES-D. Correlations of magnitude less than 0.10 are unlikely to be of theoretical or clinical interest.

## Results

Clinical-demographic characteristics of the 2,225 NSHS95 participants who contributed data to the current analyses are reported in Table 1. The mean age of the sample was 48.2±18.9 years, and approximately 50% of the sample was female. The mean leukocyte telomere length for the overall cohort was 5,301±587 base pairs, and the mean total score on the CES-D was 7.4±7.9. Based on a cutpoint of ≥10 for the CES-D overall score, 27.6% of participants had elevated depressive symptoms; based on a cutpoint of ≥16 for the CES-D overall score, 12.1% of participants had a probable depressive disorder.

**Table 1:** Clinical-demographic characteristics of 2,225 participants in the 1995 Nova Scotia Health Survey (NSHS95) study.

Clinical-demographic characteristic	Value
Age, years	48.2 (18.9)
Female, *n* (%)	1,110 (49.9)
Smoking, *n* (%)	586 (26.3)
Body mass index, kg/m^2^	27.0 (5.5)
Diabetes mellitus, *n* (%)	101 (4.5)
Systolic blood pressure, mm Hg	126.0 (17.7)
Diastolic blood pressure, mm Hg	76.9 (9.6)
Low-density lipoproteins, nmol/l	3.3 (0.9)
High-density lipoproteins, nmol/l	1.3 (0.3)
Use of lipid-lowering medications, *n* (%)	42 (1.9)
Use of aspirin, *n* (%)	90 (4.0)
Framingham risk score points	2.1 (9.2)
Previous ischemic heart disease, *n* (%)	160 (7.2)
Telomere length, base pairs	5,301 (587)
CES-D total score (Range: 0–52)	7.4 (7.9)
Probable depressive disorder (CES-D≥16), *n* (%)	269 (12.1)
Elevated depressive symptoms (CES-D≥10), *n* (%)	613 (27.6)
CES-D depressed mood subscale score (Range: 0–20)	2.0 (3.2)
CES-D motor impairment subscale score (Range: 0–20)	3.6 (3.5)
CES-D lack of well-being subscale score (Range: 0–12)	1.5 (2.2)
CES-D interpersonal difficulties subscale score (Range: 0–6)	0.3 (0.8)

Values are means (standard deviation) except where noted otherwise.

Abbreviations. CES-D, Center for Epidemiologic Studies—Depression scale

**Figure 1:**
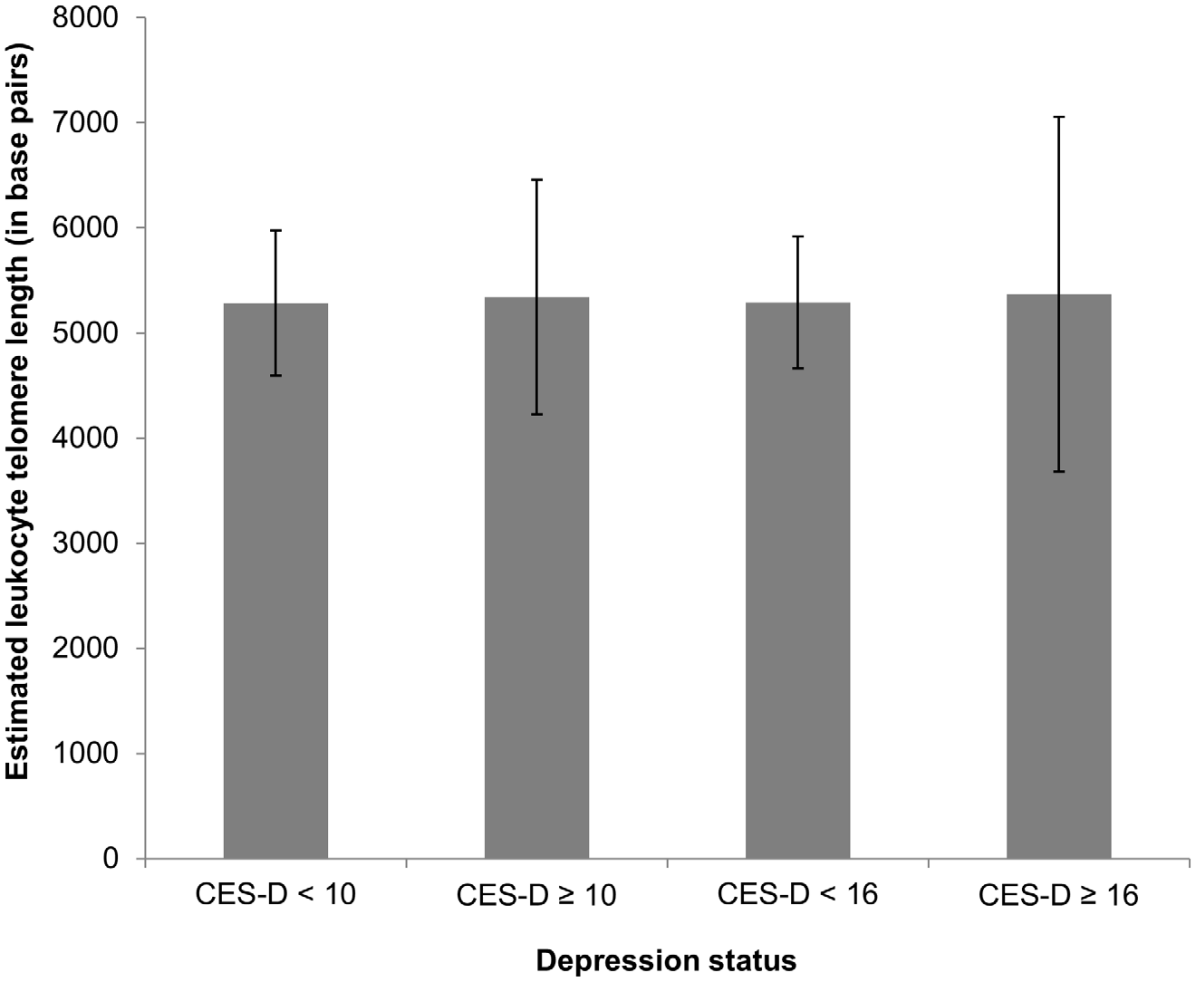
Estimated mean leukocyte telomere length by depression status.

Estimated mean leukocyte telomere lengths (in base pairs) by depression status are shown in Figure 1. Depressive symptoms (B = 27.6 base pairs per standard deviation increase in CES-D, 95% confidence interval [CI] = 3.1–52.1, *p* = 0.027), elevated depressive symptoms (CES-D≥10: B = 59.6, 95% CI = 5.1–114.2, *p* = 0.032), and probable depressive disorder (CES-D≥16: B = 77.1, 95% CI = 2.3–151.9, *p* = 0.043) were each associated with *longer* leukocyte telomere length in unadjusted linear regression models. However, after adjustment for age and sex, these relations were no longer statistically significant (Table 2): depressive symptoms (B = 9.5 base pairs per standard deviation increase in CES-D, 95% CI = −14.6–33.6, *p* = 0.44), elevated depressive symptoms (CES-D≥10: B = 20.8, 95% CI = −32.6–74.3, *p* = 0.45), and probable depressive disorder (CES-D≥16: B = 50.2, 95% CI = −22.8–123.1, *p* = 0.18) were not significantly associated with leukocyte telomere length. Results were similar in models that additionally adjusted for Framingham risk score, BMI, and previous ischemic heart disease. Neither depressive symptoms nor probable depressive disorder interacted with age, sex, or BMI categories in the prediction of leukocyte telomere length in fully adjusted models (all *p's*≥0.10). Results of analyses that excluded participants with ischemic heart disease in the five years prior to the baseline interview were similar to results of the primary analyses. Specifically, depressive symptoms (B = 30.8 base pairs per standard deviation increase in CES-D, 95% CI = 5.1–56.5, *p* = 0.019), elevated depressive symptoms (CES-D≥10: B = 64.9, 95% CI = 8.1–121.7, *p* = 0.025), and probable depressive disorder (CES-D≥16: B = 68.7, 95% CI = −9.5–147.0, *p* = 0.085) were associated with leukocyte telomere length in unadjusted linear regression models, but the association became non-significant in the adjusted models (all *p's*≥0.26).

**Table 2:** Associations between depressive symptoms, probable depressive disorder (CES-D≥16), elevated depressive symptoms (CES-D≥10), and leukocyte telomere length.

Adjusted for	Unstandardized regression coefficients predicting telomere length in base pairs, B (95% CI)					
	Depressive symptoms a	*P*	CES-D≥16	*P*	CES-D≥10	*P*
–	27.6 (3.1–52.1)	0.027	77.1 (2.3–151.9)	0.043	59.6 (5.1–114.2)	0.032
Age and sex	9.5 (-14.6–33.6)	0.44	50.2 (-22.8–123.1)	0.18	20.8 (-32.6–74.3)	0.45
Age, sex, FRS	10.0 (-14.1–34.1)	0.42	51.4 (-21.6–124.3)	0.17	22.1 (-31.4–75.6)	0.42
Age, sex, FRS, BMI	9.3 (-15.1–33.7)	0.46	49.5 (-24.3–123.2)	0.19	22.5 (-31.5–76.6)	0.41
Age, sex, FRS, BMI, previous IHD	11.1 (-13.4–35.6)	0.37	52.8 (-20.9–126.6)	0.16	25.8 (-28.3–79.9)	0.35

Abbreviations: CES-D, Center for Epidemiologic Studies—Depression scale; FRS, Framingham risk score; BMI, body mass index; IHD, ischemic heart disease

a Regression coefficients represent the predicted number of base pairs by which leukocyte telomeres increase *per standard deviation increase* on the CES-D.

Secondary analyses of the association between depressive symptom clusters and leukocyte telomere length were also all non-significant. The continuous depressed affect, somatic concerns, positive affect, and interpersonal difficulties subscales of the CES-D each failed to significantly predict leukocyte telomere length in either unadjusted or adjusted linear regression models (all *p*'s≥0.17). Further, high versus low scores on each of these subscales (defined as the fourth quartile versus the first quartile) were not associated with leukocyte telomere length (all *p*'s≥0.10).

## Discussion

In this sample of 2,225 apparently healthy participants from a population-based study, we found that neither depressive symptoms nor probable depressive disorder were significantly associated with leukocyte telomere length after adjustment for possible confounding factors including age, sex, BMI and other cardiovascular risk factors. These findings, based on one of the largest cohorts to date in which the telomere-depression association has been examined, fail to replicate those of several previous studies. In addition, this study is among the first to examine the association of leukocyte telomere length with specific depressive symptom clusters, an important aim given the heterogeneity of the depression phenotype [17] and the differential associations of depressive symptom clusters with biological [19], [20] and clinical factors [18]. However, we did not observe an association between leukocyte telomere length and any of the specific depressive symptom clusters included in the current study.

It has been hypothesized that the increased risk of CVD associated with depressive symptoms is in part

attributable to shortened telomeres—perhaps via increased levels of oxidative stress or inflammation or decreased levels of antioxidant enzymes [5]. Our findings show that depression is not associated with shorter leukocyte telomere length in this population-based sample. Although we did not consider cardiovascular endpoints in the current analyses or directly test a mediational model linking depression to cardiovascular outcomes via telomere length, our failure to find an independent association between depression and leukocyte telomere length suggests that telomere length may not play an important role. Indeed, the single study that directly tested whether premature leukocyte shortening contributed to the excess morbidity and mortality associated with depression in patients with stable coronary heart disease found that adjusting for baseline leukocyte telomere length in depressed patients did not affect the association between depression and cardiovascular prognosis [5].

Several previous studies have found that psychosocial factors, including depressive disorders and depressive symptoms, are associated with shorter leukocyte telomere length. Simon and colleagues [4], for instance, reported that leukocyte telomere length was significantly shorter in a clinical sample of 44 individuals with chronic mood disorders—assessed by using a structured clinical interview—compared to 44 non-psychiatric, age-matched control participants. These findings were consistent for the smaller subset of patients with MDD (*n* = 15), and persisted in analyses adjusted for chronological age, sex, and smoking history. Lung and colleagues [14] likewise documented an association between depression and shortened leukocyte telomere length among 253 patients with psychiatrist-diagnosed MDD from a hospital in Taiwan compared to a stratified, random household sample of 411 community controls. Hartmann and colleagues also reported significantly shorter telomere length in a non-randomly selected group of 54 inpatients with diagnoses of MDD compared to 20 healthy controls [13]. Differences between these previous studies and the current one are that the previous studies were not population-based and were relatively small compared to the current study, thereby limiting the generalizability of their reported results. These previous studies also considered a limited set of confounding factors in adjusted analyses, and restricted their definition of depression to MDD. Finally, other studies that have examined the associations among leukocyte telomere length and depression were limited to patients with prevalent cardiovascular disease (e.g., patients with stable ischemic heart disease [5] or heart failure [38]). The participants in our study were from a relatively large and well-characterized population-based cohort, with a wide range of depressive symptoms and CVD risk. Further, the sample was of sufficient size to detect small but important associations between depressive symptoms and telomere length, and our analyses considered important covariates that may be associated with shorter telomere length. Therefore, the lack of association between depressive symptoms and telomere length in our study may be generalizable to a broader population.

Although significant findings have been reported, as described above, not all studies have found an association between depression and leukocyte telomere length. Wolkowitz and colleagues failed to observe an association between leukocyte telomere length in 18 untreated participants with MDD compared to 17 apparently healthy controls [16]. In addition, Surtees and colleagues did not find evidence of an association between leukocyte telomere length and lifetime MDD, past-year MDD, or depressive symptoms among 4,441 women aged 41 to 80 years from the population-based UK European Prospective Investigation into Cancer (EPIC)-Norfolk study [15]. However, they used a self-assessment approach to assessing MDD that included restricted criteria from the *Diagnostic and Statistical Manual of Mental Disorders* (DSM-IV), and depressive symptoms were assessed with the Short -Form 36 (SF-36), which is more often used as a measure of quality of life than a measure of depression.

The absence of an association between concurrent depressive symptoms and leukocyte telomere length may reflect the fact that leukocyte telomere length is influenced by cumulative environmental factors over time, whereas assessments of depressive symptoms generally focus on only the past one-to-two weeks. Indeed, although Wolkowitz and colleagues failed to find an association of leukocyte telomere length with MDD or depressive symptoms, telomere length was inversely correlated with lifetime depression exposure, after controlling for age [16]. Specifically, the average telomere length of outpatients who were both depressed and had a high lifetime depression exposure (i.e., greater than the median exposure) was significantly shorter than the average telomere length of healthy controls who were individually matched to cases by sex, ethnicity, and age. Studies of the association between other psychosocial stressors and leukocyte telomere length have likewise suggested the importance of considering cumulative exposure to psychological stress. In a study by Epel and colleagues, for instance, greater chronicity of caregiving stress (in years), but not caregiving status itself, was associated with shorter telomere length [12]. Our study did not include a measure of the chronicity or lifetime duration of depressive symptoms. Similarly, we did not have an assessment of early life trauma, which some researchers have hypothesized as contributing to the association between shorter telomere length and depression reported in previous studies [13]. Indeed, some studies have linked shorter leukocyte telomere length to childhood maltreatment or other adverse childhood experiences among individuals without major psychiatric disorders [39] and among those with posttraumatic stress disorder [40]. However, not all studies have replicated this association [41].

Our study is among the first to consider the association among leukocyte telomere length and specific depressive symptom clusters, and found that none of the specific depressive symptom clusters were associated with telomere length. We have previously shown that depression is a complex and occasionally chronic cluster of diseases [18], and other authors have likewise discussed limitations of the broad depression phenotype and proposals for disaggregating its components [17]. Indeed, some studies suggest that specific depressive symptom clusters (e.g., somatic symptoms) are associated with cardiac prognosis while others (e.g., cognitive symptoms) are not. Other research has found differential associations between specific depressive symptom clusters and C-reactive protein [19], heart rate variability, spontaneous baroreflex sensitivity, and the cortisol awakening response [20]. Although no single scale assesses all possible depressive symptoms or depressive symptom clusters, our findings based on the CES-D do not support differential associations between specific depressive symptom clusters and telomere length, and thus do not support telomere length as a mediator between depressive symptom clusters and cardiac risk in this population.

The current study has several limitations. First, generalizability of the current results to other populations may be limited given that the NSHS95 cohort was relatively culturally and ethnically homogeneous (consisting of nearly entirely of white participants). Second, we only assessed depressive symptoms and did not use structured diagnostic interviews or physician diagnoses of depressive disorders. However, our analyses did examine the association of elevated depressive symptoms and probable depressive disorder with leukocyte telomere length using two cutpoints on the CES-D that have acceptable sensitivity and specificity in relation to MDD [23], [24] and/or predictive validity with respect to ischemic heart disease [25]. Third, our data are cross-sectional, and we do not have repeat assessments of either leukocyte telomere length or of depressive symptoms. As such, we could not examine life-time burden of depressive symptoms. In addition, different methods of measuring leukocyte telomere length may have impacted our findings. Some researchers have concluded that the PCR method shows good agreement with the Southern blot method [42], while others have suggested limitations of the precision of PCR-based assessments of telomere length [43]. Previous studies of depression and leukocyte telomere length have used either PCR-based methods [15], [16] as in our study or Southern blot-based methods [4], [13], [14]. To our knowledge, no prior study has directly compared the various methods of measuring leukocyte telomere length with respect to their associations with depression, and this issue deserves consideration in future research. Finally, we did not measure telomerase levels, which one previous study has shown as being significantly related to depressive symptom severity [44].

Notwithstanding these limitations, the results of this large, representative, population-based study, which was adequately powered to detect small but important associations between depressive symptoms and leukocyte telomere length, suggest that concurrent depressive symptoms are not associated with shorter leukocyte telomere length. In addition, elevated depressive symptoms, probable depressive disorder, and specific depressive symptom clusters were all unrelated to leukocyte telomere length in this sample. Future research should further investigate whether chronicity of depression or early life trauma are associated with telomere length.
